# Whole-body PET acceptance test in 2D and 3D using NEMA NU 2-2001 protocol

**DOI:** 10.4103/0971-6203.37479

**Published:** 2007

**Authors:** Shamurailatpam Dayananda Sharma, R. Prasad, Bina Shetye, V. Rangarajan, D. Deshpande, S. K. Shrivastava, K. A. Dinshaw

**Affiliations:** Department of Medical Physics, Tata Memorial Hospital, Mumbai, Maharashtra, India; * Department of Bio-Imaging Unit, Tata Memorial Hospital, Mumbai, Maharashtra, India; ** Department of Radiation Oncology, Tata Memorial Hospital, Mumbai, Maharashtra, India

**Keywords:** National Electrical Manufacturers Association NU2 - 2001, performance measurement, PET/CT

## Abstract

Integrated PET/CT has emerged as an integral component of oncology management because of its unique potential of providing both functional and morphological images in a single imaging session. In this work, performance of the ‘bismuth germinate (BGO) crystal’-based PET of a newly installed Discovery ST PET/CT was evaluated in 2D and 3D mode for whole-body scanning using National Electrical Manufacturers Association (NEMA) NU 2-2001 protocol and the recommended phantoms. During the entire measurements, the system operates with an energy window of 375-650 keV and 11.7 ns coincidence time window. The set of tests performed were spatial resolution, sensitivity, scatter fraction (SF) and counting rate performance. The average transaxial and axial spatial resolution measured as full width at half maximum (FWHM) of the point spread function at 1 cm (and 10 cm) off-axis was 0.632 (0.691) and 0.491 (0.653) cm in 2D and 0.646 (0.682) and 0.54 (0.601) cm in 3D respectively. The average sensitivity for the two radial positions (*R* = 0 cm and *R* = 10 cm) was 2.56 (2.63) cps/kBq in 2D and 11.85 (12.14) cps/kBq in 3D. The average scatter fraction was 19.79% in 2D and 46.19% in 3D. The peak noise equivalent counting rate (NECR) evaluated with single random subtraction was 89.41 kcps at 49 kBq/cc in 2D and 60 kcps at 12 kBq/cc in 3D acquisition mode. The NECR with delayed random subtraction was 61.47 kcps at 40.67 kBq/cc in 2D and 45.57 kcps at 16.45 kBq/cc in 3D. The performance of the PET scanner was satisfactory within the manufacturer-specified limits. The test result of PET shows excellent system sensitivity with relatively uniform resolution throughout the FOV, making this scanner highly suitable for whole-body studies.

Positron emission tomography (PET) integrated to multi-slice computed tomography (CT) (PET/CT) has emerged as an integral component of oncology management in the recent past, due to its unique ability to provide both functional and morphological information in a single imaging session. In this increasingly popular dual-modality tomography, Florine-18 fluro-2-deoxyglucose (F-18 FDG) PET provides functional information due to preferential uptake and retention of glucose by tumors, and CT provides the anatomic details and attenuation-correction data to PET. The integration of PET to CT not only enhances PET image quality but also reduces total scan time by almost half and avoids the need for a posterior alignment by the use of co-registration algorithms. Besides its wide applications in the diagnosis of various solid cancers, F-18 FDG-PET/CT is increasingly used in radiation therapy planning for staging, tumor volume delineation, treatment response evaluation and recurrence detection.[1]

The PET image quality is degraded by several physical factors, including scatter, random events, attenuation, dead time and noise. While some can be corrected, others depend on the performance of scanner. Detectors are the most critical components of a PET scanner. Various models of PET, either standalone or integrated to multi-slice CT, make use of different scintillators such as Bismuth Germanate (BGO), Lutetium Oxyorthosilicate (LSO), Gadolinium Oxyorthosilicate (GSO) and different detector architecture like blocks of detectors and continuous pixilated detectors. The performance of this newly emerging dual-modality tomography needs to be evaluated at the time of installation and periodically thereafter to ensure optimum system performance in a reproducible and reliable manner according to accepted protocols. PET performance was characterized following National Electrical Manufacturers Association (NEMA) NU 2-1994 protocol.[2] However, increasing practice of whole-body scanning in oncological applications in the recent past has led to the formation of newer NEMA NU 2-2001 protocol.[3] This updated protocol accounts for the performance test of whole-body PET both in two and three dimensions, which was not addressed in the previous protocol NU 2-1994 (N-94). In this work, performance of the PET component of our newly installed Discovery ST PET/CT was evaluated following NEMA NU 2-2001 (N-01) protocol.

## Materials and Methods

The first integrated PET/CT of the country, Discovery ST (GE Medical Systems, USA), was installed in the Bio-Imaging Unit of our hospital in November 2004 and has been in clinical use since January 2005. The Discovery ST (D-ST) consists of one integrated gantry containing (a) 16-slice, slip-ring design CT X-ray tube and Hi-light matrix II detector assembly, (b) ‘BGO crystal’-based PET and (c) a common imaging table with the provision of flat carbon fiber table top for radiotherapy application.

### System description

**Figure 1:**
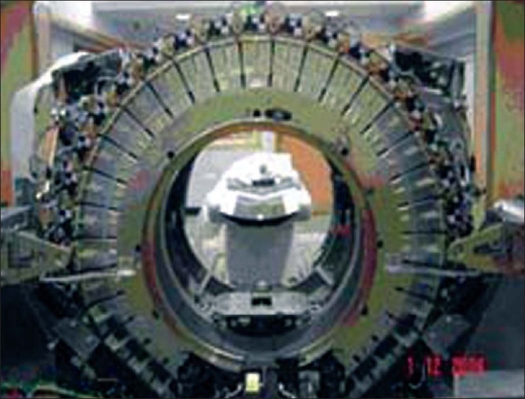
Arrangement of detector modules in D-ST PET rings

The PET in D-ST consists of 24 detector rings comprising 12,096 BGO crystals. Each crystal is of size 0.63 × 0.63 × 3 cm^3^ and is organized in blocks. Each block, containing 6 × 6 crystals, is coupled to a single photomultiplier tube with four anodes and constitutes a module, which is then arranged in the 24 rings. It allows 47 images to be obtained per bed position, spaced by 0.327 cm and covering an axial field of view (FOV) of 15.7 cm. The arrangement of modules in detector rings is shown in Figure 1. The image acquisition can be carried out either in 2D or 3D mode by inserting or retracting the tungsten septa of 0.8 cm thickness and 5.4 cm length. In both acquisition modes, the system operates with an energy window of 375-650 keV and 11.7 ns coincidence time window. Mechanical design integration provides a wide 70 cm bore, 88.6 cm ring diameter, short tunnel length and compact scanner design. PET is also equipped with an auto-loading pin radioactive source of ^68^Ge (55.5 MBq) for system calibration and daily quality control.

**Figure 2:**
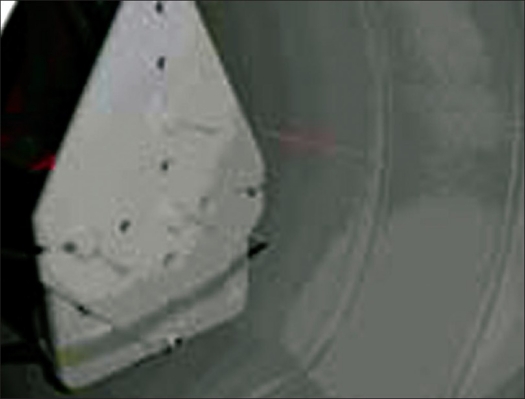
Experimental setup for spatial resolution measurement. Three ^18^F point sources contained in capillary tubes are placed at *X* = 0 cm, *Y* = 1 cm; *X* = 0 cm, *Y* = 10 cm; and *X* = 10 cm, *Y* = 0 cm in the center of the axial FOV of the scanner

Attenuation correction is based entirely on CT numbers to PET attenuation transformation factors. Image reconstruction in 2D mode can be performed with either filtered backprojection (FBP) or ordered-subset expectation maximization (OSEM), whereas the 3D image reconstruction supports both 3D reprojection and Fourier rebinning (FORE) followed by either FBP or a weighted least squares (WLS) OSEM iterative reconstruction. Both 2D and 3D iterative reconstructions include attenuation compensation within the model to more accurately preserve the statistical nature of the input data. Scatter correction is calculated with the Bergstrom convolution in 2D and an analytic model-based technique in 3D. Random correction can be conducted with delayed-event coincidence measurements or from an estimate of randoms generated from the crystal singles rate.

### NEMA 2001 (N-01) measurements

The set of tests performed under N-01 using recommended phantoms were (i) spatial resolution, (ii) sensitivity, (iii) scatter fraction (SF) and counting rate performance.

*Spatial resolution*: It represents the ability of the system to distinguish between two points of radioactivity in an image. Three ^18^F point sources (<0.1 cm extending in any direction), each having activity concentration of 185 MBq/cc (5 mCi/cc), were prepared and contained in capillary tube with an inner diameter (ID) less than 0.1 cm. The capillary tubes were aligned parallel to the long axis of the tomography and distributed such that point sources were placed at the locations corresponding to *X* = 0 cm, *Y* = 1 cm; *X* = 0 cm, *Y* = 10 cm; and *X* = 10 cm, *Y* = 0 cm in the center of the axial FOV of the scanner as shown in Figure 2. Data were acquired in 2D and 3D modes with each acquisition set for 60 s. The images were reconstructed using the FBP (2D) and FORE followed by FBP (3D) algorithm into a 256 × 256 matrix with a ramp filter and a 0.63-cm cutoff. The reconstruction FOV was set to 25 cm and centered at *X* = 5 cm, *Y* = −5 cm. Spatial resolution was determined from the reconstructed 2D and 3D data by measuring the full width at half maximum (FWHM) and tenth width at half maximum (TWHM) of the point spread functions in all three directions through the peak of the activity distribution in the three orthogonal directions.

**Figure 3:**
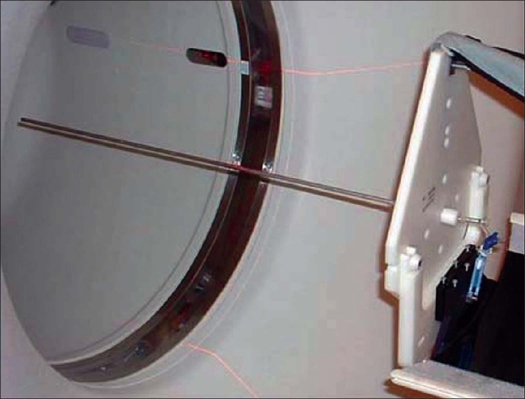
Experimental setup for sensitivity measurement. A line source surrounded by aluminum tubes of known thickness is suspended at the center of the scanner FOV

*Sensitivity*: The sensitivity of a scanner represents its ability to detect annihilation radiation. In the N-01 standard, the absolute sensitivity of a scanner was measured as the coincidence event rate per unit radioactivity (cps/MBq) from a sufficiently low activity line source suspended within the scanner FOV in the absence of attenuating media. A uniform line source was prepared by filling with 9.6 MBq of ^18^F in a 70-cm long plastic tube having 0.31 cm inner diameter (ID) and 5.2 ml volume. This line source after inserting in a 70-cm long concentric aluminum tubes having attenuation coefficient of 0.09965/cm and inner diameter (ID) of 0.39 cm was suspended at the center of the scanner FOV as shown in Figure 3. Measurements (cps) were made both in 2D and 3D acquisition modes. Each data acquisition was set for duration of 1 min. Similar measurements (cps) were performed after successively adding aluminum tubes of bigger ID (0.7, 1.02, 1.34 and 1.66 cm) one above the other. Extrapolation of the response to zero absorber thickness gives an attenuation-free estimate of sensitivity. To evaluate the sensitivity of the scanner at another position of FOV, the whole measurement was repeated at a radial distance of 10 cm from the center.

**Figure 4:**
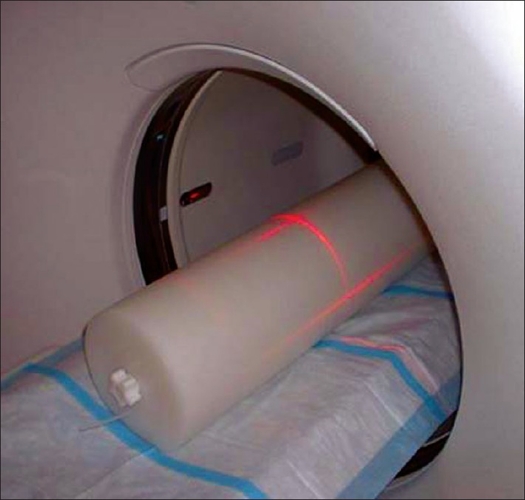
Experimental setup for scatter fraction (SF) and count rate (CR) performance test. Cylindrical polyethylene phantom (20 cm diameter and 70 cm length) with the line source inserted posteriorly was positioned at the center of scanner FOV

*Scatter fraction (SF) and count rate (CR) performance*: The intrinsic scatter fraction is a measure of the relative system sensitivity to scatter. The scatter fraction (SF) is defined as the ratio of scattered events to total events, which are measured at a sufficiently low counting rate so that random coincidences, dead-time effects and pileup are negligible. Total events are the sum of unscattered events (trues) and scattered events. For the measurement of SF and count rate (CR) performance, a line source was prepared by filling with 2.59 GBq of ^18^F in the same 70 cm long plastic tube. It was then inserted into the hole located at a radius of 4.5 cm off the central axis of the polyethylene cylindrical phantom (20 cm diameter and 70 cm length). This phantom, with the line source posterior, was positioned at the center of scanner FOV [Figure 4] and imaged repeatedly over a period of 12 h. Data was acquired in 2D and 3D modes and recorded without delayed-event randoms data. The SF was then measured from the reconstructed data according to the N-01 standard and plotted for each slice across the axial FOV of the scanner. The average system SF was also calculated. The CR performance of the scanner was evaluated using all data acquisition time points. The total system counting rate; trues, randoms and scatter event rates; and noise equivalent counting rates (NECR) were calculated and plotted versus the activity concentration in the plastic tube. Peak values and corresponding activity concentration for these rates were also determined according to the NU-01 standard.

## Results

**Figure 5:**
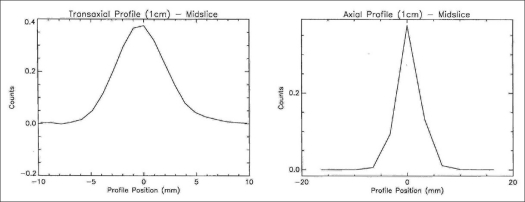
Axial and transverse profiles for a point source at 1 cm off center measured in 3D following NEMA NU 2-2001 protocol

**Figure 6:**
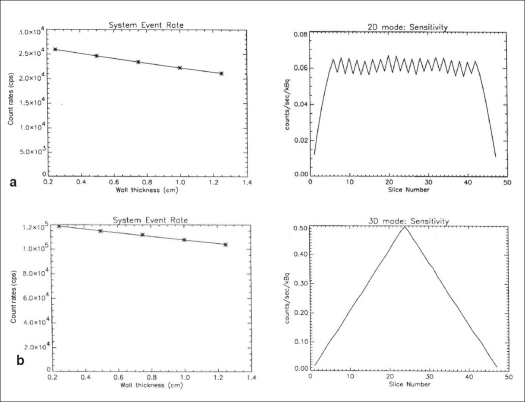
Sensitivity across axial FOV measured in 2D (Figure 6a) and 3D (Figure 6b) according to NEMA NU 2-2001 protocol

**Figure 7:**
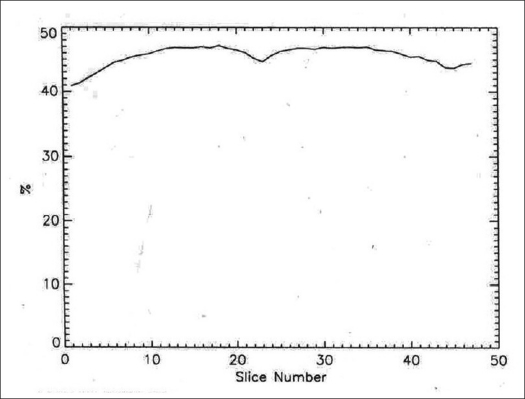
Scatter fraction in 3D mode across axial FOV of scanner measured according to NEMA NU 2-2001 protocol

**Figure 8:**
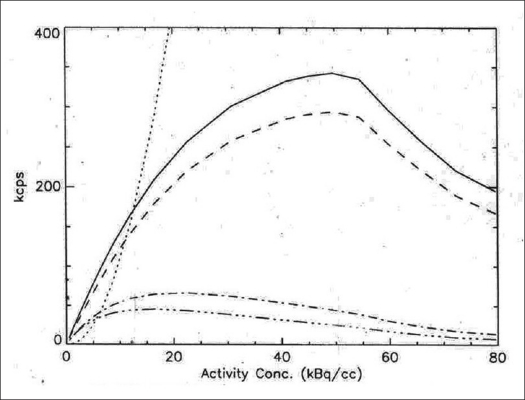
Counting rate performance of PET scanner at various levels of activity within phantom. Data acquired in 3D mode following NEMA NU 2-2001 protocol. Trues - solid; Randoms - dotted; Scatter - dashed; NECR (1R) - dash dot; NECR (2R) - dash dot dot

The 2D transaxial (average of radial and tangential) and axial profiles for a point source at 1 cm off center are shown in Figure 5, while Table 1 represents the 2D and 3D average transaxial and axial spatial resolution expressed as FWHM and FWTM of the point source located at 1 and 10 cm radial position. The average of the transaxial and axial spatial resolution measured as FWHM at 1 cm off axis was 0.561 cm in 2D and 0.593 cm in 3D. Both the 2D and 3D resolutions were degraded at 10 cm off axis and were 0.672 and 0.641 cm respectively. Figure 6 shows the 2D and 3D sensitivity profile across the available FOV of the scanner. The results of the sensitivity test in 2D and 3D acquisition configurations at both radial locations (R = 0 and R = 10 cm) are shown in Table 2. The system has an average sensitivity of 2.56 and 2.63 cps/kBq in 2D for the two radial positions R = 0 and R = 10 cm respectively. Sensitivity in 3D was approximately 4.6 times higher compared to that in 2D. The results for the SF and CR tests in 3D mode are shown in Figures 7 and 8 respectively; whereas Table 3 summarizes the values of the count rate of the system and the corresponding activity concentration at the peak true rate, peak random rate, peak scatter rate and peak NECR (*k* = 1) and NECR (*k* = 2). The average scatter fraction across the axial FOV of the scanner was 19.79% in 2D and 46.19% in 3D. For the 2D data acquisition mode, the peak true rate was 309.29 kcps and occurred at 122.66 kBq cc, whereas the peak random rate was 1303.58 kcps at 134.83 kBq/cc. The peak noise equivalent counting rate (NECR) evaluated with single random subtraction (*k* = 1 in NEC formula) was 89.41 kcps at 49 kBq/cc in 2D and 60 kcps at 12 kBq/cc in 3D, whereas the NECR with delayed random subtraction (*k* = 2 in NEC formula) was 61.47 kcps at 40.67 kBq/cc in 2D and 45.57 kcps at 16.45 kBq/cc in 3D acquisition mode.

**Table 1:** Transaxial and axial resolution of a point source in 2D and 3D kept at different radial positions

*Radial position of point source*	*FWHM in cm*				*FWHM in cm*			
	
	*Transaxial*		*Axial*		*Transaxial*		*Axial*	
			
	*2D*	*3D*	*2D*	*3D*	*2D*	*3D*	*2D*	*3D*
*R* = 1 cm	0.632	0.646	0.491	0.540	1.271	1.237	1.078	1.232
*R* = 10 cm	0.691	0.682	0.653	0.601	1.338	1.289	1.343	1.399

Transaxial - average of radial and tangential; FWHM - full width at half maximum; FWTM - full width at tenth maximum

**Table 2:** Sensitivity in 2D and 3D acquisition modes

*Sensitivity (cps/kBq)*	*2D*	*3D*
*R* = 0 cm	2.565	11.854
*R* = 10 cm	2.627	12.137

**Table 3:** Counting rate performance in 2D and 3D acquisition modes

*Parameters*	*Kilo counts per second (kcps) @ kBq/cc*	
	
	*2D*	*3D*
Peak true rate	309.29 @ 122.66	342.51@ 49.61
Peak random rate	1303.58 @ 134.84	2443.66 @ 79.64
Peak scatter rate	76.32 @ 122.66	293.86 @ 49.61
Peak NEC (*k* = 1R) rate	89.41 @ 49	60 @ 12
Peak NEC (*k* = 2R) rate	61.47 @ 40.67	45.57 @ 16.45

## Discussion

Continuous efforts have been made to standardize the performance measurement of PET scanner. In 1991, a task group from the society of nuclear medicine (SNM) published a set of measurements.[4] Shortly the National Electrical Measurements Association (NEMA) formed a committee and refined the SNM test, resulting in the formation of NU 2-1994 (N-94) protocol.[2] N-94 protocol assumes axial FOV of all scanners to be lesser than 17 cm and supports only two-dimensional imaging for a 20 cm diameter and 20 cm long phantom. Thus N-94 protocol is more suitable to assess the performance of the PET scanners in conditions comparable to those of neurological studies. Since the publication of N-94 protocol, there have been several developments in PET scanner technology. In the last few years, three-dimensional whole-body ^18^F-FDG study has become the prominent type of PET study performed by most centers. Moreover, the modern PET scanners have axial FOV as large as 25 cm. To respond to this changing technology, a new protocol NU 2-2001(N-01) was published in 2001.[3] In N-01, 70 cm long phantom is used to account for the activity contribution from outside the FOV, and tests are defined for both 2D and 3D acquisition configurations. This document specifies procedures for acquiring and analyzing test data using standard phantoms and sources. Margaret *et al*. described the details of the development of different protocols and advantages of following N-01 protocol over N-94.[5] An inter-laboratory comparison study conducted in Austria to assess the image quality of 85% of all their PET (dedicated; D-PET and coincidence camera CC-PET) scanners using NEMA-2001 demonstrated considerable differences not only between CC-PET and D-PET systems but also between individual D-PET systems, with possible consequences for clinical interpretation of images and measurement of quantitative indices such as the standardized uptake value.[6] The findings also strongly demonstrate the necessity for carrying out regular quality control programs for this ‘new imaging system’-following standard protocols.

The measured (FWHM) and manufacturer-specified spatial resolution in both 2D and 3D acquisition modes agree within ±3% (mean) at 1 and 10 cm radial position. Spatial resolution reported in the literatures varies depending on the type and dimension of the crystal size.[5–9] Our data is comparable with other's finding from the similar PET scanner with same crystal dimension.[8,9] The system sensitivity in both 2D and 3D acquisition modes was better than the manufacturer-specified value and data reported from similar PET.[8,9] The measured average scatter fraction in both acquisition modes was ≈5% more than the manufacturer-specified values. The noise equivalent counting (NEC) peak rate measured at 49 kBq/cc using *k* = 1R in 2D mode was 6.4% higher than the manufacturer-specified value. On the contrary, peak NEC (*k* = 2R) at 12 kBq/cc in 3D was 4.8% lesser than the manufacturer-specified values. This variation could be due to the variation in the activity concentration level within the scanner FOV during the data acquisition. The 2D and 3D NEC peak rate for *k* = 1R reported from the similar PET scanner with same crystal dimension is 90.2 and 67.8 kcps at 52.5 and 12 kBq/cc respectively.[8] Image quality test has not been performed due to the non-availability of NEMA/IEC 2000 Torso phantom.

## Conclusion

The performance of Discovery ST PET/CT evaluated using NEMA-2001 protocol and recommended phantoms satisfies the manufacturer-recommended specifications both in 2D and 3D acquisition modes. The test result of PET shows excellent system sensitivity with relatively uniform resolution throughout the FOV, making this scanner highly suitable for whole-body studies.

## Acknowledgment

We would like to express our gratitude to Mr. Krishna Toraskar of Wipro GE Medical System for his technical assistance.
